# The microbiome exists in the neuroretina and choroid in normal conditions and responds rapidly to retinal injury

**DOI:** 10.3389/fopht.2025.1719090

**Published:** 2025-12-09

**Authors:** Xuexue Cui, Jinyan Qi, Caijiao Yi, Jian Liu, Xiang-Ling Yuan, Wen Deng, Heping Xu

**Affiliations:** 1 AIER Eye Institute, Changsha Aier Eye Hospital, Changsha, Hunan, China; 2 AIER Academy of Ophthalmology, Central South University, Changsha, Hunan, China

**Keywords:** inflammation, wound healing, *Faecalibacterium*, *Bifidobacterium*, neuroretina

## Abstract

**Purpose:**

To investigate the microbial profiles in the retina and RPE/choroid, and how they respond to retinal injury.

**Methods:**

Adult C57BL/6J mice were subjected to retinal laser burns using a photocoagulator. One and 24h later, the retina and RPE/choroid were collected under strict sterile conditions and processed for 16S rRNA paired-end sequencing (2×250). The data were analyzed using R software, GraphPad Prism, OmicShare, and Wekemo Bioincloud.

**Results:**

Microbiota were detected in the retina and RPE/choroid under normal physiological conditions. The alpha diversity was higher in the retina than in the RPE/choroid. All retinal microbiotas at the phylum level and 12 out of 14 at the genus level were shared with those of RPE/choroid. The top phyla were *Firmicutes*, *Proteobacteria*, and *Actinobacteria*. Retinal laser injury reduced the alpha diversity but did not affect beta diversity. In the RPE/choroid, the abundance of *Actinomyces* and *Roseburia* decreased, and the abundance of *Lactobacillus* increased significantly after laser injury. The abundance of *Sphingomonas* in the retina decreased, and the abundance of *Faecalibacterium* and *Bifidobacterium increased (P<0.05)* after laser injury in the retina. *Faecalibacterium* and *Bifidobacterium* are positively linked to Th17/IL-17 signaling and RIG-I-like receptor signaling pathways, as well as antigen processing and presentation.

**Conclusions:**

The neuroretina and RPE/choroid have diverse microbiomes under normal conditions. Their richness and evenness are relatively stable in the retina compared to those in the RPE/choroid. Retinal laser injury enriches *Faecalibacterium* and *Bifidobacterium* in ocular tissues, and these microbiotas may participate in retinal wound healing through modulating inflammation.

## 1 Introduction

The human body harbors trillions of commensal bacteria that have coevolved with the host, constituting a functionally integrated ecosystem that is now under active investigation (1). Gut dysbiosis, characterized by altered microbial diversity and richness, drives the pathogenesis of metabolic disorders, autoimmunity, cardiovascular disease, and neurodegeneration (2–5). Growing evidence links gut dysbiosis to ocular pathologies, including age-related macular degeneration (AMD), uveitis, diabetic retinopathy (DR), and glaucoma (6–9). For instance, fecal samples from AMD patients exhibit enrichment of *Anaerotruncus, Oscillibacter, Ruminococcus torques*, and *Eubacterium ventriosum* (10), and a higher ratio of *Firmicutes/Bacteroidetes* (11). In primary open-angle glaucoma, increased abundance of *Prevotellaceae, Enterobacteriaceae*, and *Escherichia coli* established causal links to optic neurodegeneration (12). Beyond the gut, next-generation sequencing has revealed microbial communities on the ocular surface and within intraocular tissues (13). Dysbiosis of these communities has been linked to keratitis (14, 15), dry eye disease (16), and keratoconus (13). Mechanistically, microbiota imbalance may contribute to the pathogenesis of human disease through modulating immune response either systemically or locally within the disease tissue (17–19).

Apart from the body surface (skin and mucosal surfaces), microbes have also been detected in body fluids, including blood (20, 21), cerebrospinal fluid (22, 23), and intraocular fluid (24) from healthy and diseased individuals. Emerging evidence suggests that microbes also exist inside the tissues, such as the brain, in people with Alzheimer’s disease (25), the retina of WT and diabetic (Akita) mice (26), the placenta (27), and the breast tissue (28, 29). The mammalian eye has long been considered an immune-privileged organ, anatomically and functionally protected from microbial colonization by physical barriers, i.e., the blood-retinal barrier (BRB) and local immune regulatory mechanisms (30). However, emerging evidence challenges this dogma, suggesting that even highly specialized tissues, such as the retina, may harbor microbial communities (26). The intraocular microbiome may modulate retinal immune response and participate in disease development (31, 32). We previously reported that retinal pigment epithelial (RPE) cells and vascular endothelial cells express high levels of an antimicrobial peptide (AMP), lysozyme, which may protect the retina from blood-borne pathogens (33, 34). The concept of a tissue-associated microbiome has transformed our understanding of immune regulation, metabolic homeostasis, and disease susceptibility in organs such as the gut, skin, and lung (35–39). Yet, the presence and role of microbial populations in the retina and choroid and their responses to retinal injury remain largely unexplored.

Understanding whether a local microbiome exists in the posterior eye segment and its potential function is of considerable significance. The retina and choroid are highly vascularized and metabolically active tissues that maintain a delicate balance between immune tolerance and activation. Perturbations in this balance are implicated in major blinding diseases such as AMD and DR. If local microbial populations contribute to immune homeostasis or metabolic signaling, their alteration could represent a previously unrecognized driver of retinal pathology.

Here, we characterized the microbial profiles in the retina and RPE/choroid using 16S rRNA sequencing and further investigated their response to retinal laser injury. We found that both tissues harbor diverse microbiomes under normal conditions, and that the microbial profile is rapidly altered after injury.

## 2 Materials and methods

### 2.1 Animals

C57BL/6J mice (6–8 weeks of age) were purchased from SJA Laboratory Animal Co., Ltd. (Changsha, Hunan, China), housed under specific pathogen-free (SPF) conditions with a 12/12 h light-dark cycle and libitum access to standard chow and water. All experimental protocols complied with the Association for Research in Vision and Ophthalmology (ARVO) Statement for the Use of Animals in Ophthalmic and Vision Research and received ethical approval from the Animal Care and Use Committee of AIER Eye Institute (Approval ID: AEI20230048).

### 2.2 Induction of retina injury

We used retinal laser burns as a retinal injury model. The animals were anesthetized with isoflurane using an inhalation system. Induction was performed with 3–4% isoflurane in oxygen, followed by maintenance at 1.5–2% isoflurane. The depth of anesthesia was monitored throughout the procedure. Pupils were dilated with 0.5% tropicamide/phenylephrine (Santen Pharmaceutical Co., Ltd., Osaka, Japan). Ocular surface hydration was maintained with carboxymethylcellulose sodium (Allergan Pharmaceutical Co., Ltd., Dublin, Ireland). Four laser burns (200 mW, 100 ms, 60 μm spot) were applied 2 optic disc diameters away from the optic nerve head using a 532-nm photocoagulator (Topcon, Tokyo, Japan) as detailed in previous studies (40–42).

### 2.3 Tissue collection for 16S rRNA analysis

At the end of the experiment, animals were euthanized using a carbon dioxide (CO_2_) chamber, followed by cervical dislocation to confirm death, in accordance with institutional and international ethical guidelines. Retina and RPE/choroid were collected from normal non-lasered, 1h, and 24h post-laser injury mice (n = 8 mice per group). Mice that underwent anesthesia without retinal injury served as controls. All procedures were performed under Class II biosafety cabinet conditions. The eyeballs were enucleated under sterile conditions and treated immediately with 10% betadine for 3 min, followed by thorough washes with 70% ethanol and irrigation with sterile saline. Sterile cotton swabs were utilized to sample the globe surface before and after the globe cleaning procedure for 16S rRNA analysis as procedure controls. The globes were dissected with autoclaved instruments. The anterior segment was removed, and the retina and RPE/choroid were collected using sterile forceps, placed into nuclease-free vials, snap-frozen in liquid nitrogen, and stored at −80^0^C for 16S rRNA sequencing. Additionally, 10% betadine, 70% ethanol, sterile saline, and lysis buffer were subjected to 16S rRNA sequencing as extraction and PCR amplification controls. Mixtures of the known bacteria *Akkermansia muciniphila* and *Faecalibacterium prausnitzii* were used as positive controls.

### 2.4 DNA extraction and polymerase chain reaction amplification

Genomic DNA isolation was performed using the QIAamp DNA Stool Mini Kit (Cat: 51504, Qiagen, Hilden, Germany) following the manufacturer’s instructions. For microbial community profiling, the V3-V4 hypervariable segments of bacterial 16S rRNA genes were amplified with universal primers 341F: CCTACGGGNGGCWGCAG and 806R: GGACTACHVGGGTATCTAAT incorporated with sample-specific barcodes (8-nt length). The PCR products were purified (Axygen Biosciences, Silicon Valley, USA), and quantified fluorometrically (QuantiFluor-ST, Promega, Madison, USA). Equimolar concentrations of purified amplicons were combined with Illumina-based paired-end sequencing (250 bp read length). The raw reads from the mouse retina and RPE/choroid have been deposited in the NCBI SRA database (access code PRJNA1158449).

### 2.5 Bioinformatics and statistical analysis of 16S-rRNA sequencing data

Sequence data underwent stringent quality control, excluding reads with >10% ambiguous bases (N) or <80% high-quality bases (Q≥20). Paired-end reads were assembled into preliminary tags via FLASH (v1.2.11; 10bp overlap threshold, 2% mismatch tolerance). Subsequent noise reduction was performed using QIIME (v1.9.1) to obtain refined sequences. For Amplicon Sequence Variant (ASV) construction, demultiplexed raw data (demux plugin) underwent adapter trimming (cutadapt) before DADA2-based processing (quality filtering, denoising, read merging, and chimera elimination). Taxonomic classification was executed in QIIME2 with reference to SILVA (16S/18S) and UNITE (ITS) databases. Taxon abundances were computed via custom Perl scripts and rendered graphically in SVG format.

### 2.6 Statistical analysis

Statistical analyses were conducted in GraphPad Prism 8 (GraphPad Software, San Diego, CA), with significance thresholds set at p<0.05. Microbial alpha diversity was evaluated via six ecological metrics (species richness estimators: Chao1/ACE; diversity indices: Shannon/Simpson; sequencing depth: Good-coverage) processed through QIIME2, with subsequent visualizations created in R. Beta diversity patterns were examined through PCoA based on Jaccard and unweighted UniFrac distance matrices, complemented with functional prediction via PICRUSt2, all implemented within the R environment.

For graphical representations, comparative analyses (bar plots, Venn diagrams) and microbial abundance heatmaps were constructed using OmicShare’s web-based tools (https://www.omicshare.com/tools). Interaction networks were modeled via Wekemo Bioincloud (https://www.bioincloud.tech). Non-parametric comparisons across groups were conducted using the Kruskal-Wallis test with Dunn’s *post-hoc* correction. Quantitative data were expressed as mean ± SD.

## 3 Results

### 3.1 Microbial composition in the retina and RPE/choroid under normal conditions

Microbial 16S rRNA (V3-V4) high-throughput sequencing revealed the presence of numerous microbial taxa in the retina and RPE/choroid (n = 8). Rarefaction curves indicated adequate sequencing depth for microbial diversity capture (**Supplementary Figure S1A**). 16S DNA agarose gel electrophoresis showed clear DNA products from the retina and RPE/choroidal samples in all groups (**Supplementary Figure S1B**). The control samples, including sterile cotton swabs sampled before and after cleaning the global surface, lysis buffer, 10% betadine, sterile saline, and ethanol used for cleaning the global, did not show any bands in agarose gel electrophoresis (**Supplementary Figure S1B**). The results suggest that the microbial taxa detected in the retina and RPE/choroid were not due to contamination from sample preparations.

**Figure 1:**
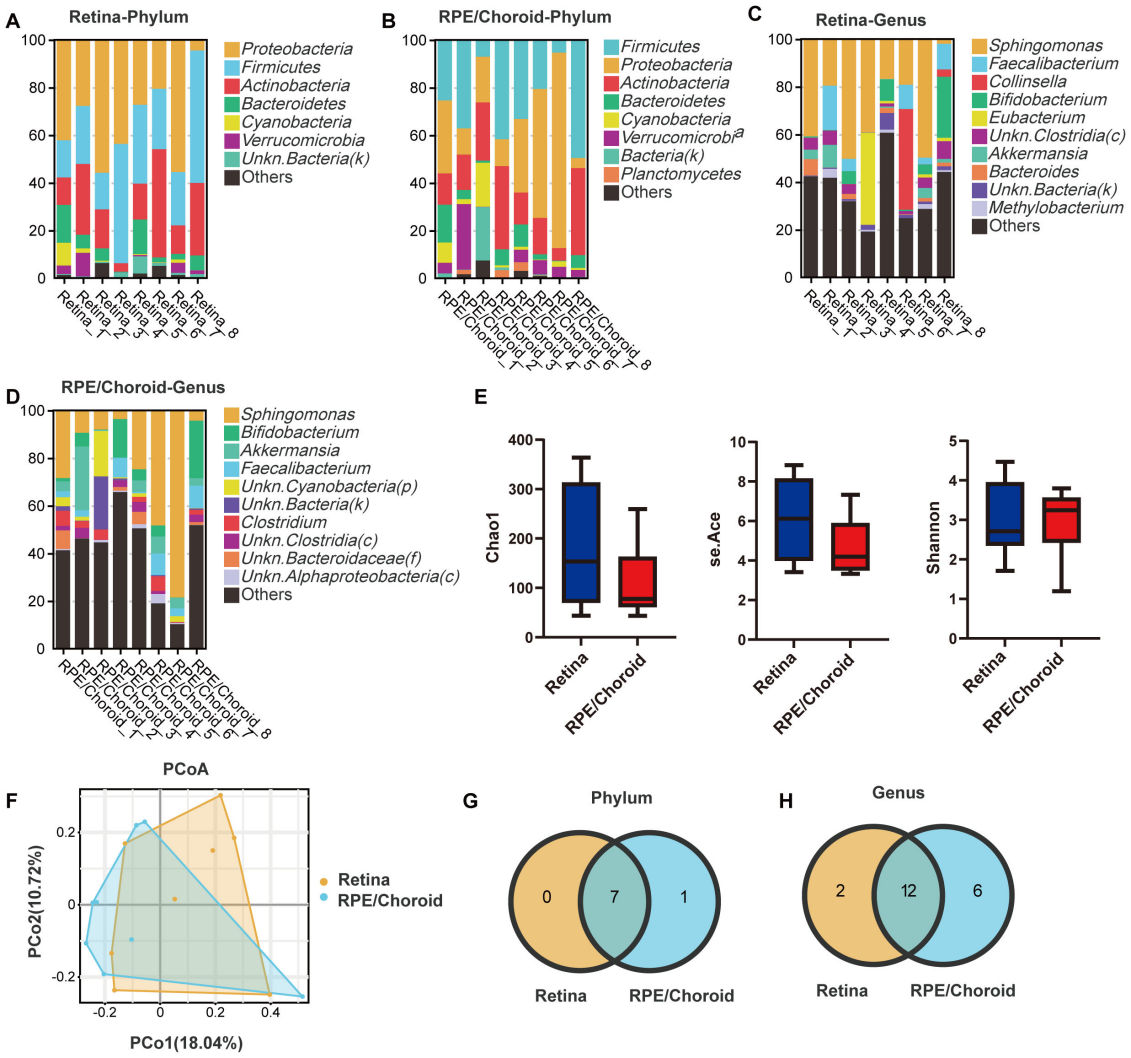
Taxonomic profiling and diversity metrics in healthy retina and RPE/choroid. **(A, B)** Bar plot depicting the taxonomic composition at the phylum level in the retina **(A)** and RPE/choroid **(B)**. **(C, D)** Bar plot depicting the taxonomic composition at the genus level in the retina **(C)** and RPE/choroid **(D)**. Microbial taxa were determined using the criteria of presence in >50% of the samples and an average abundance above 1% across all samples. n=8. **(E)** Box plots showing the α-diversity, specifically, the Chao1, se.Ace, and Shannon indices in different samples. Kruskal-Wallis with Dunn’s multiple comparisons test. **(F)** PCoA elucidates the similarities and differences between different healthy samples. **(G, H)** The number of unique or shared microbiota at the level of phylum **(G)** and genus **(H)** between two healthy tissues. Overlapping areas show the number of shared microbiotas.

Under normal conditions, a total of 7 and 8 phyla and 14 and 18 genera were identified from the retina and RPE/choroid, respectively. The top three phyla were *Proteobacteria* (relative abundances: retina = 34.9%, RPE/choroid = 30.5%), *Firmicutes* (relative abundances: retina = 30.2%, RPE/choroid = 27.7%), and *Actinobacteria* (relative abundances: retina = 20.4%, RPE/choroid = 19.4%) (**Figures 1A, B**). The top three genera were *Sphingomonas, Faecalibacterium*, and *Collinsella* in the retina (relative abundance: 29.7%, 6.0%, 5.7%), *Sphingomonas, Bifidobacterium*, and *Akkermansia* in the RPE/choroid (relative abundance: 25.7%, 7.2%, 6.3%) (**Figures 1C, D**).

Although the retina exhibits higher alpha diversity (Chao1/se.Ace index) than the RPE/choroid (**Figure 1E**), the difference is not statistically significant, indicating potentially a greater microbial community resilience. The beta diversity showed no significant difference between the two tissues (**Figure 1F**), suggesting similar overall taxonomic composition. Indeed, all retinal microbiotas at the phylum level (**Figure 1G**) and 12 out of 14 at the genus level (**Figure 1H**) are shared with those of RPE/choroid. The shared microbiotas at the genus level include *Sphingomonas, Faecalibacterium, Bifidobacterium, Unkn. Clostridia(c), Akkermansia, Blautia, Bacteroides, Clostridium, Unkn. Eubacteriales(o), Unkn. Actinobacteria(p), Methylobacterium*, and *Unkn. Bacteroidaceae(f)*. The relative abundance of these shared microbiotas did not differ between the retina and the RPE/choroid.

Our results suggest the presence of microbiota in RPE/choroid and the neuronal retina, and the microbial composition is similar between the two tissues.

### 3.2 Retinal injury alters microbial profiles and composition

**Figure 2:**
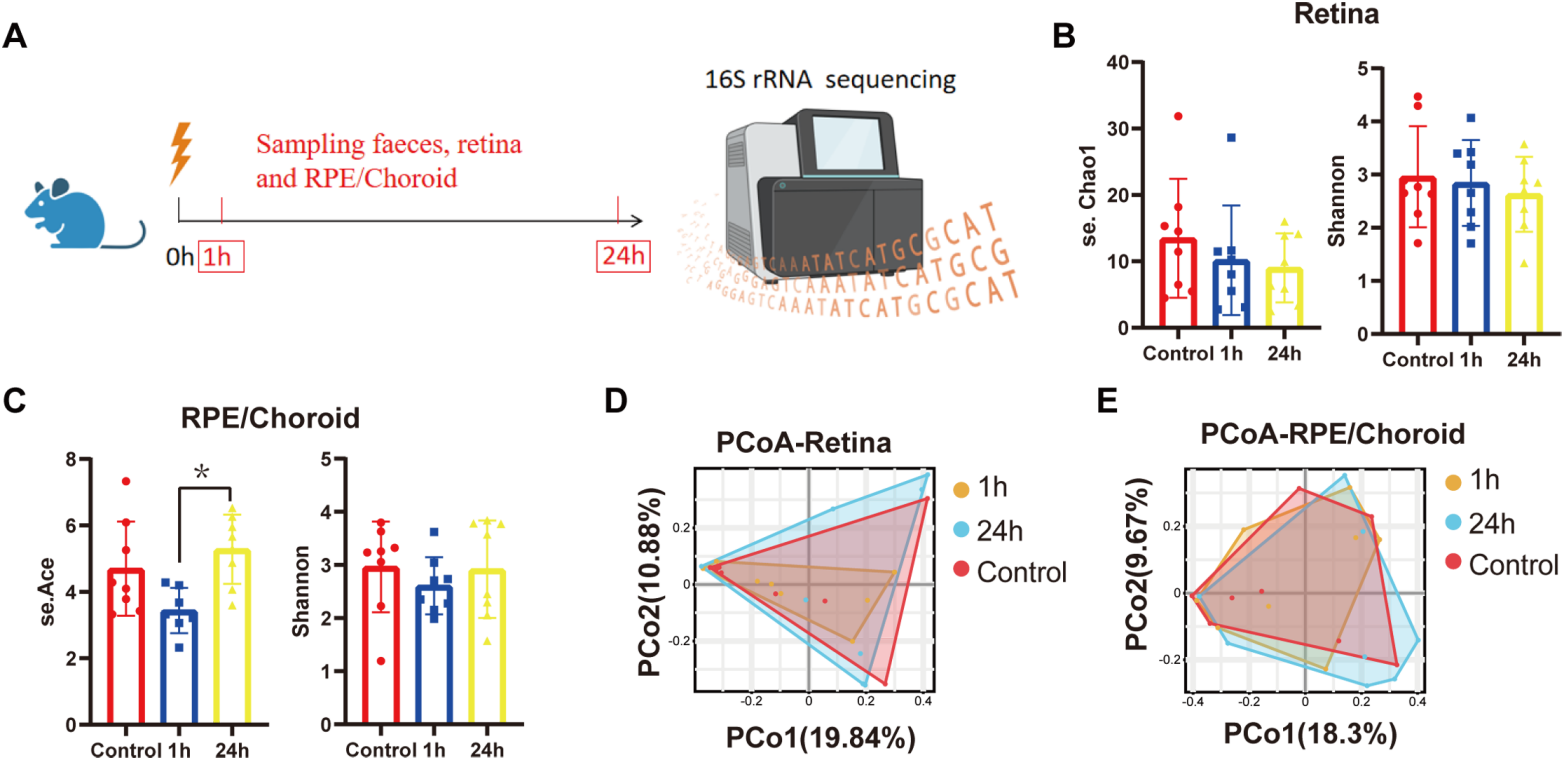
Distribution of microbiota in the retina and RPE/choroid following retinal laser injury. **(A)** Diagram showing the experimental design. Mice were treated with retinal laser burns. Retina and RPE/choroid were collected at 1h and 24h later. The difference of α-diversity in the retina **(B)** and RPE/choroid **(C)** at different times post-laser injury. *p<0.05, n=8. Kruskal-Wallis with Dunn’s multiple comparisons test. Retinal laser injury-induced PCoA changes in the retina **(D)** and RPE/choroid **(E)** at different times post-laser injury.

To understand the microbial response to retinal injury, we profiled microbiota in the retina and RPE/choroid at 1h and 24h post-injury using 16S rRNA sequencing (**Figure 2A**). The α-diversity of the retinal samples showed no significant changes after laser injury compared with the sham-operated group (**Figure 2B**). Interestingly, the se.Chao1 index revealed a significant increase in α-diversity in RPE/choroid samples at 24h compared to 1h post-laser injury (**Figure 2C**). The β-diversity showed no significant differences in the retina and RPE/choroid at different times after laser injury (**Figures 2D, E**). Our results suggest that retinal laser injury had a limited impact on the richness and evenness of intraocular microbiota within 24 h.

**Figure 3:**
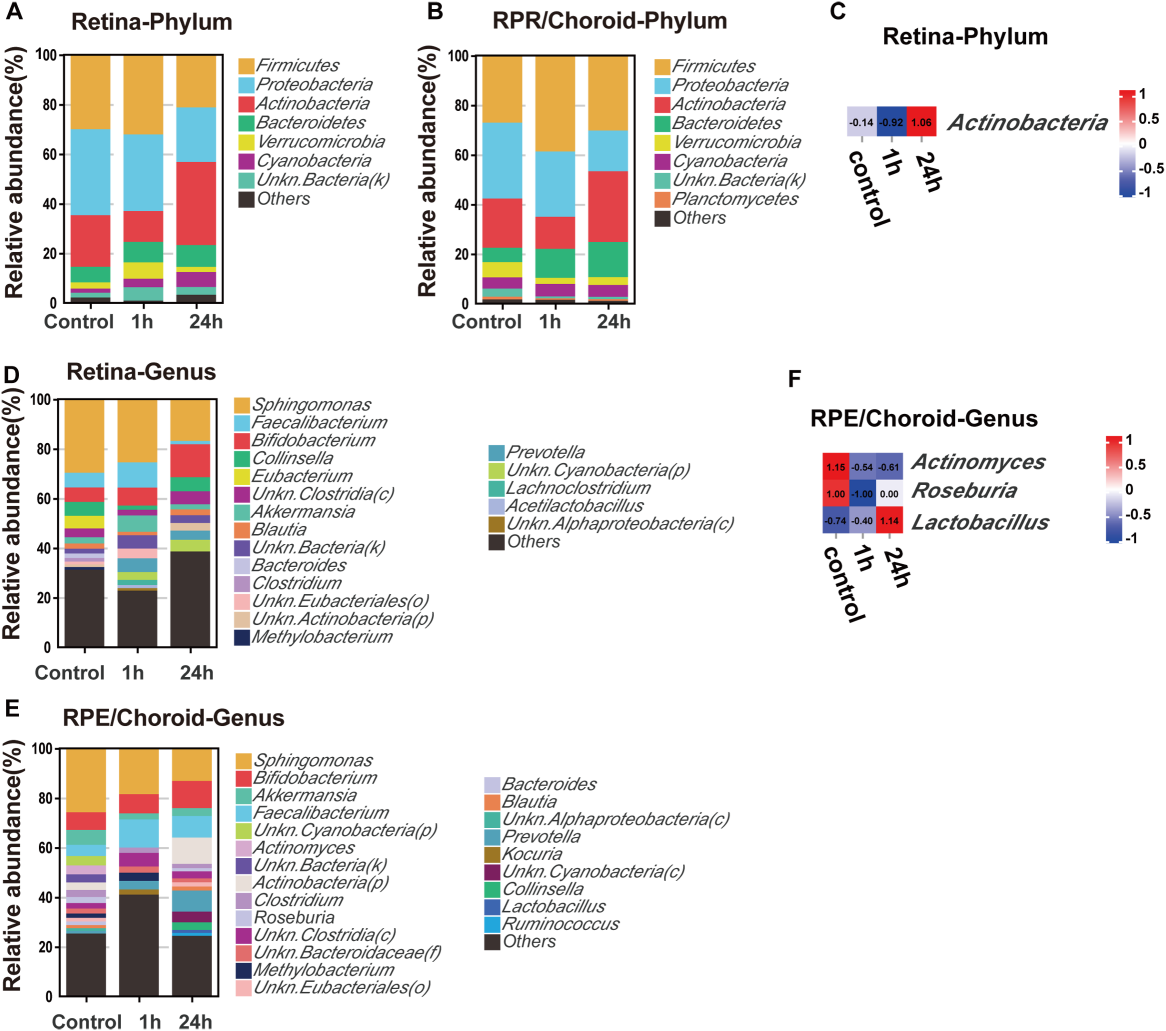
Alterations in the composition and relative abundance of tissue microbiota after retinal injury. Top 10 Taxonomic distributions of bacteria at the phylum level in the retina **(A)** and RPE/choroid **(B)** at different times after retinal injury. **(C)** Heatmaps showing altered microbiota in the retina at different times post-retinal injury at the phylum level. Taxonomic distributions of bacteria at the genus level in the retina **(D)** and RPE/choroid **(E)** at different times after retinal injury. **(F)** Shifts at the genus-level microbiota in the RPE/choroid at different times after retinal injury.

The taxonomy of each tissue at different times was assessed independently at the phylum and genus levels. At the phylum level, *Firmicutes, Proteobacteria, Actinobacteria, Bacteroidetes*, and *Verrucomicrobia* were enriched in all tissues (**Figures 3A, B**). The abundance of *Actinobacteria* was significantly increased in the retina at 24h compared to 1h post-laser injury (*p = 0.04*, **Figure 3C**). No significant change was detected in the RPE/choroid.

At the genus level, the retina and RPE/choroid exhibited a high abundance of *Sphingomonas, Faecalibacterium*, and *Bifidobacterium* (**Figures 3D, E**). The abundance of *Sphingomonas* decreased over time after laser injury (**Figures 3D, E**). In the RPE/choroid, the abundance of *Actinomyces* and *Roseburia* decreased, while *Lactobacillus* increased significantly after laser injury (**Figure 3F**). No significant change was detected in the retina.

**Figure 4:**
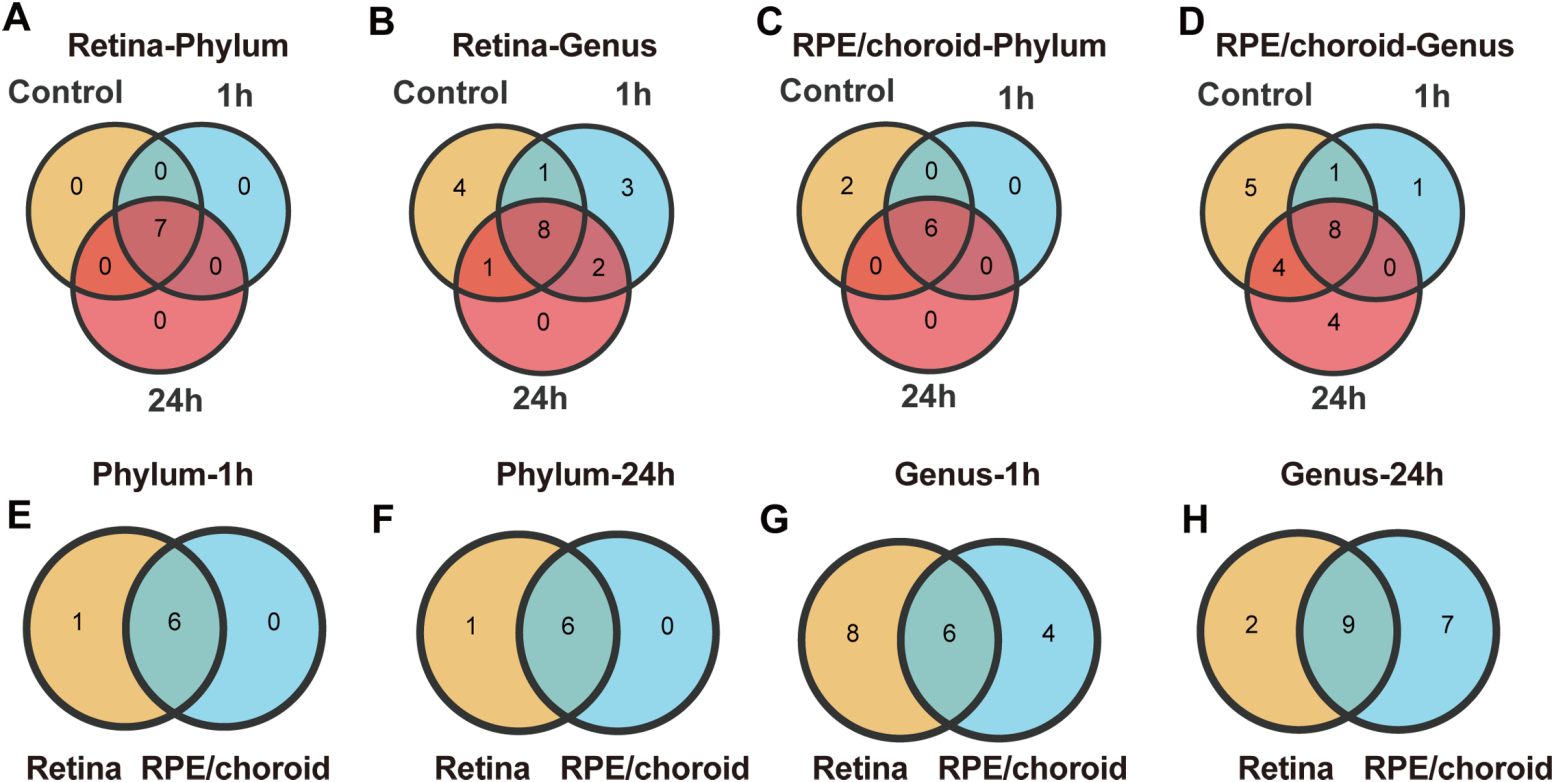
The shared and unique microbiota in the retina and RPE/choroid with/without retinal laser injury. **(A-D)** Venn analysis of the shared microbiota at different times. Overlapping areas show the number of shared microbiotas. **(A, B)** The number of shared or unique microbiota at the level of phylum **(A)** and genus **(B)** in the retina at different times. **(C, D)** The number of shared or unique microbiota at the level of phylum **(C)** and genus **(D)** in RPE/choroid at different times. **(E-H)** Venn analysis of the shared microbiota at different tissues. **(E-F)** The number of shared or unique microbiota in the level of phylum between retina and RPE/choroid at 1h **(E)** and 24h **(F)** post-retinal laser injury. **(G-H)** The number of shared or unique microbiota at the genus level between retina and RPE/choroid at 1h **(G)** and 24h **(H)** post-retinal laser injury.

Next, we examined the shared and unique microbiota at different timepoints from the same tissue (present in at least 50% of the samples and average abundance ≥ 1%). A total of 7 phyla and 19 genera were identified in the retina. All phyla (*Firmicutes, Proteobacteria, Actinobacteria, Bacteroidetes, Verrucomicrobia, Cyanobacteria*, and *Unkn. Bacteria(k)*) and 8 (*Bifidobacterium, Faecalibacterium, Collinsella, Blautia, Akkermansia, Sphingomonas, Unkn. Clostridia(c)*, and *Unkn. Bacteria(k))* out of 19 genera were shared by samples from different times (**Figures 4A, B**). Three genera (*Unkn. Alphaproteobacteria(c), Acetilactobacillus, Lachnoclostridium*) exclusively present in the retinas from 1h post-laser injury (**Figure 4B**), suggesting a rapid microbial alteration.

Eight phyla and 23 genera were detected in the RPE/choroid. Six phyla (*Firmicutes, Proteobacteria, Actinobacteria, Bacteroidetes, Verrucomicrobia*, and *Cyanobacteria*) and 8 genera (*Sphingomonas, Bifidobacterium, Akkermansia, Faecalibacterium, Clostridium, Unkn. Clostridia(c), Unkn. Bacteroidaceae(f)*, and *Prevotella*) were shared by samples from different times (**Figures 4C, D**). One (*Methylobacterium*) and 4 (*Lactobacillus, Collinsella, Ruminococcus, Unkn. Cyanobacteria(c)*) were unique to 1h and 24h samples, respectively (**Figures 4C, D**).

We further identified 6 shared microbiotas (*Firmicutes, Proteobacteria, Cyanobacteria, Bacteroidetes, Verrucomicrobia*, and *Actinobacteria*) between the retina and RPE/choroid at the phylum level (**Figures 4E, F**) at 1h and 24h post-retinal injury. At the genus level, we identified 6 shared microbiotas (*Bifidobacterium, Faecalibacterium, Akkermansia, Prevotella, Sphingomonas, Unkn. Clostridia(c)*) at 1h post-retinal injury and 9 shared microbiotas (*Bifidobacterium, Faecalibacterium, Collinsella, Blautia, Akkermansia, Unkn. Clostridia(c), Prevotella*, and *Sphingomonas*) at 24h (**Figures 4G, H**). The number of shared microbiotas in the two tissues significantly decreased after retinal injury at the genus level (**Figures 4E–H**, compared with those under normal conditions, **Figures 1E, F**).

The presence of numerous shared microbiotas between the retina and RPE/choroid and at different times after laser injury indicates potential functional and compositional interdependence across these ocular regions under different conditions.

### 3.3 Predicted pathways of microbiome with and without retinal laser injury

**Figure 5:**
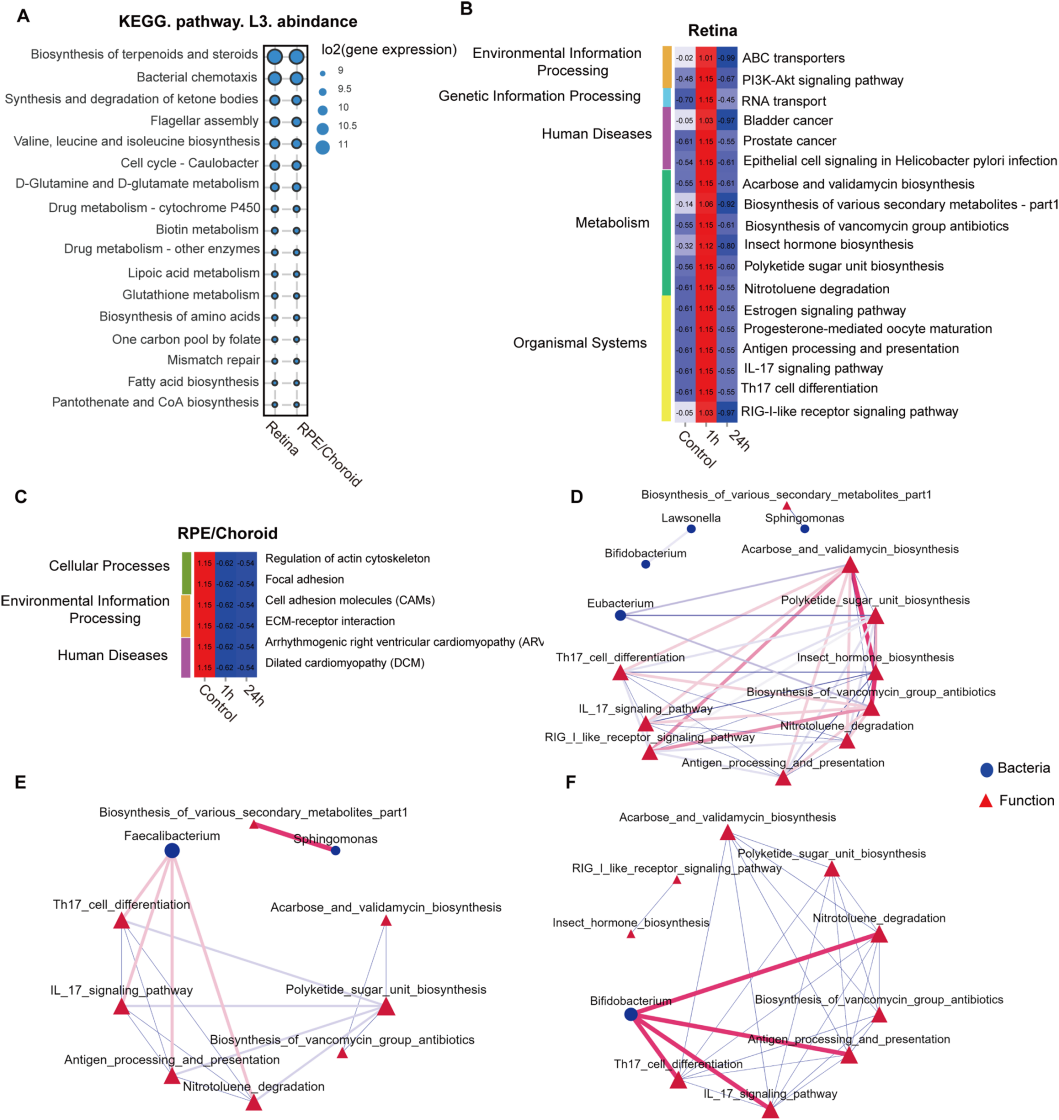
Retinal injury-induced changes in the predicted microbiota functional pathways. **(A)** Top 15 average abundance of KEGG level-3 pathways in the retina, RPE/choroid and feces under normal conditions. KEGG level-3 pathways at different times in the retina **(B)** and RPE/choroid **(C)**. **(D-F)** Network analysis of the link of bacteria communities (top 30 genera) with the predicted pathways in the retina under normal conditions **(D)**, 1h **(E)**, and 24h after retinal laser injury **(F)**. The red line between nodes indicates a positive correlation, and the blue line between nodes indicates a negative correlation. The size of the nodes represents the degree of a genus of bacteria or KEGG-level 3 pathways.

To understand the biological significance of the retinal and RPE/choroidal microbiota, we employed the PICRUSt2 platform to predict microbial functions. KEGG level 3 analysis revealed that, under normal conditions, pathways related to biosynthesis of terpenoids and steroids and bacterial chemotaxis were enriched in both retina and RPE/choroid (**Figure 5A**). At 1h post-laser injury, 18 pathways were significantly upregulated in the retina, including 6 metabolism pathways and 4 immune system-related pathways (antigen processing and presentation; IL-17 signaling pathway; RIG-I-like receptor signaling pathway; Th17 cell differentiation) (**Figure 5B**). In contrast, six pathways were significantly downregulated in the RPE/choroid after injury (**Figure 5C**), encompassing functional associations with environmental and genetic information processing, human diseases, metabolism, organismal systems, and cellular processes (**Figures 5B, C**).

Network analysis revealed that *Eubacterium* exhibited negative correlations with the biosynthesis of acarbose, validamycin, polyketide sugar unit, and vancomycin group antibiotics under normal conditions. Notably, acarbose/validamycin and vancomycin group antibiotics were positively associated with Th17 cell differentiation, IL-17 signaling, RIG-like-receptor signaling, and antigen processing/presentation (**Figure 5D**). *Sphingomonas*, which was detected in all tissues (**Figure 1**) and decreased following retinal injury (**Figure 3**), was negatively correlated with biosynthesis of various secondary metabolites under normal conditions; however, these associations became positive 1h after laser injury (**Figures 5D, E**). In addition, *Faecalibacterium* and *Bifidobacterium*, both abundant in the retina and RPE/choroid irrespective of injury, demonstrated positive associations with Th17 cell differentiation, IL-17 signaling pathway, antigen processing and presentation, and nitrotoluene degradation at both 1h and 24h after laser injury (**Figures 5E, F**).

Our results suggest that the intraocular microbiota might contribute to retinal health and disease by regulating the biosynthesis and degradation of molecules essential for tissue metabolism and immune function.

## 4 Discussion

Compelling evidence suggests that gut/mucus/skin microbiomes can affect human health by modulating systemic or tissue-level inflammation through metabolic products or by generating a plasma microbiome. The plasma microbiome can directly modulate circulating immune cell activation or migrate into tissues, altering the tissue microenvironment. In this study, we demonstrated the existence of microbiota in healthy retina and RPE/choroid. Many taxa are shared by the two tissues, suggesting potential functional and compositional connections across these regions. Pathway prediction revealed a potential contribution of microbiota to ocular health by maintaining metabolic balance (e.g., amino acid biosynthesis). We found that retinal laser injury altered the ocular microbial profile, and these changes can result in the upregulation of pathways associated with the biosynthesis of sugar molecules, antibiotics, and molecules related to immune function.

Previously, Deng et al. showed that aqueous humor from healthy donors harbors a unique microbiome with diverse compositions and functions (24). The authors further showed that the bacteria could be visualized by transmission electron microscopy and cultured in anaerobic conditions (24), suggesting the existence of live symbiotic bacteria inside the aqueous compartment of the human eye. Here, we detected diverse microbial compositions in the mouse retina and RPE/choroid under normal physiological conditions by 16S rRNA sequencing. We tried to culture the microbiota without any success. The top three phyla were *Firmicutes, Proteobacteria*, and *Actinobacteria.* Our results are in line with previous studies, whereby the authors reported the presence of a similar microbial signature in healthy mouse retina (26, 43). We found that the retina has higher microbial alpha diversity, indicating a stable microbial ecosystem. We previously showed that RPE cells (key cells of the outer BRB) express a variety of antimicrobial peptides (33, 34), which could restrict the entry of microorganisms from the blood circulation or the choroid into the retina. The relatively stable retinal microbial ecosystem may be related to BRB antimicrobial function and its immune-privileged nature.

Exactly how the symbiotic bacteria enter the eye remains elusive. Microbiota and their components can be carried by immune cells, such as neutrophils (44), macrophages (45) and monocytes (46) in free form from the blood or body surface (e.g., skin, mucosa, and gastrointestinal tract) to tissues, impacting local immune reactions and disease development (24, 47). Certain bacteria may also reside within autophagosomes in phagocytes and migrate to various body sites (47). Additionally, microorganisms may reside in neurons. For example, the herpes simplex virus can establish latency in the host trigeminal ganglion by remaining dormant and can be periodically reactivated under stress or immunosuppressive conditions (48). Under disease conditions, live bacteria can translocate from the gut to the eye and contribute to retinal degeneration (43). Nonetheless, the microbial beta diversity between the retina and RPE/choroid is similar, and most of the retinal taxonomic composition is shared with the RPE/choroid. This suggests that the retinal microbiome may originate from the RPE/choroid.

Upon retinal injury, the composition of the retina and RPE/choroidal microbiota changed rapidly and significantly (**Figure 3**). We recently reported that retinal injury could induce rapid changes in the gut, including altered gut microbial composition, disrupted gut epithelial barrier function, and circulating innate immune cell activation (49). This “retina-gut” axis can then feed back to the retinal wound healing process through the “gut-retina” axis (49). We found that laser injury increased the abundance of retinal *Actinobacteria* (**Figure 3**), which was significantly reduced in the gut after laser injury in our previous study (49). The laser-induced leaky gut may affect the circulating microbiome, which can then enter the RPE/choroid and retina through the damaged ocular barrier in the laser-treated eyes. These changes may modulate innate immune cell activation and contribute to retinal wound healing.

The biological significance of the ocular microbiome in retinal health remains elusive. Members of the *Actinobacteria* phylum, particularly *Bifidobacterium* spp., can synthesize the short-chain fatty acids (SCFAs) exhibiting immunomodulatory effects (50–52). *Lactobacillus* were the dominant genus in the luteolin, which could alleviate DSS-induced colitis in rats (53). The abundance of ocular *Actinobacteria* and *Lactobacillus* increased after laser injury (**Figure 3F**), and both are crucially involved in immune regulation. Previous studies reported a significant reduction in their abundance in chronic conditions, such as type 2 diabetes (54) and obesity (55), and this reduction is believed to play a role in disease progression. The laser-induced alteration of the microbiota may be the body’s attempt to repair retinal injury. This is supported by our functional analysis, which shows that the altered microbiota affects the biosynthesis of antibiotics, the PI3K-Akt signaling, and immune-related pathways such as Th17/IL-17 signaling, RIG-I-like receptor signaling, and antigen processing and presentation (**Figure 5B**). The predicted immune-related pathways are linked with *Faecalibacterium* and *Bifidobacterium* (**Figures 5E, F**), which were increased after retinal injury. *Faecalibacterium* (i.e., *Faecalibacterium prausnitzii*) has therapeutic potential in multiple diseases, including colitis (56), tumor (57), and atherosclerosis (58). They may participate in retinal wound healing through immune regulation.

Our results challenge the traditional assumption of ocular sterility by demonstrating the presence of microbiota in the retina and RPE/choroid, and their dynamic changes in disease conditions. The ocular tissue microbiome may be an additional player in the pathophysiology of retinal diseases. Further studies will be needed to determine the specific microbial profiles in each retinal disease condition. Such information will not only help uncover disease mechanisms but also develop personalized therapies (e.g., probiotics or antimicrobial peptides).

This study has several limitations. First, 16S rRNA sequencing of retina and RPE/choroidal microbiota constrained taxonomic resolution and precluded functional or quantitative microbial assessment. Second, despite extensive culturing attempts, retinal microbial viability remains undetermined. Third, the observed microbial shifts were not functionally validated. Further shotgun metagenomics may help identify species and strain-level microbes and reveal the functional potential of the microbiota. Finally, while sex differences in certain retinal diseases are well-documented (59–61) we used only males in the study. Whether the microbiota in female mice responds differently to retinal injury remains to be elucidated. Future studies will include female mice and human samples to enhance translational relevance and assess sex-specific microbiome responses. Furthermore, additional investigations are needed to elucidate the pathophysiological significance of retinal and RPE/choroidal microbiome in ocular disorders.

## 5 Conclusions

In summary, our study shows that the microbiome constitutes the retinal and RPE/choroidal ecosystem under normal physiological conditions, where it may contribute to the biosynthesis and metabolism of molecules essential for ocular homeostasis. Upon retinal injury, the microbial composition changes rapidly. These shifts might modulate retinal metabolism and intraocular immune response, thereby participating in retinal wound healing, although experimental evidence will be needed to establish the causality. A deeper understanding of the composition and function of intraocular microbiota across various disease states will help to develop better strategies to prevent or treat sight-threatening retinal diseases.

## Data Availability

The datasets presented in this study can be found in online repositories. The names of the repository/repositories and accession number(s) can be found in the article/**Supplementary Material**.

## Supplementary material

Supplementary Figure 1 Data quality control **(A)** Rarefaction curves (Sobs index) of the gut microbiota. **(B)** 16S DNA agarose gel electrophoresis of all samples. RH: retina tissue under normal condition; R1: 1h after laser injury (retina); R24: 24h after laser injury (retina); CH: RPE/choroid tissue under normal condition; C1: 1h after laser injury (RPE/choroid); C24: 24h after laser injury (RPE/choroid); 1-3:sterile cotton swabs sampled from the globe surface before cleaning procedure; 4-5,17:sterile cotton swabs sampled from the globe surface after cleaning procedure; 6-7: lysis buffer; 8-10: 10% betadine; 11-13: sterile saline; 14-16: 70% ethanol. 18-20: positive control.
