# Supplementary figures and images for: The microbiome exists in the neuroretina and choroid in normal conditions and responds rapidly to retinal injury
Source: Front Ophthalmol (Lausanne). 2025 Dec 9;5:1719090. doi: 10.3389/fopht.2025.1719090 (PMC12722843; doi:10.3389/fopht.2025.1719090)

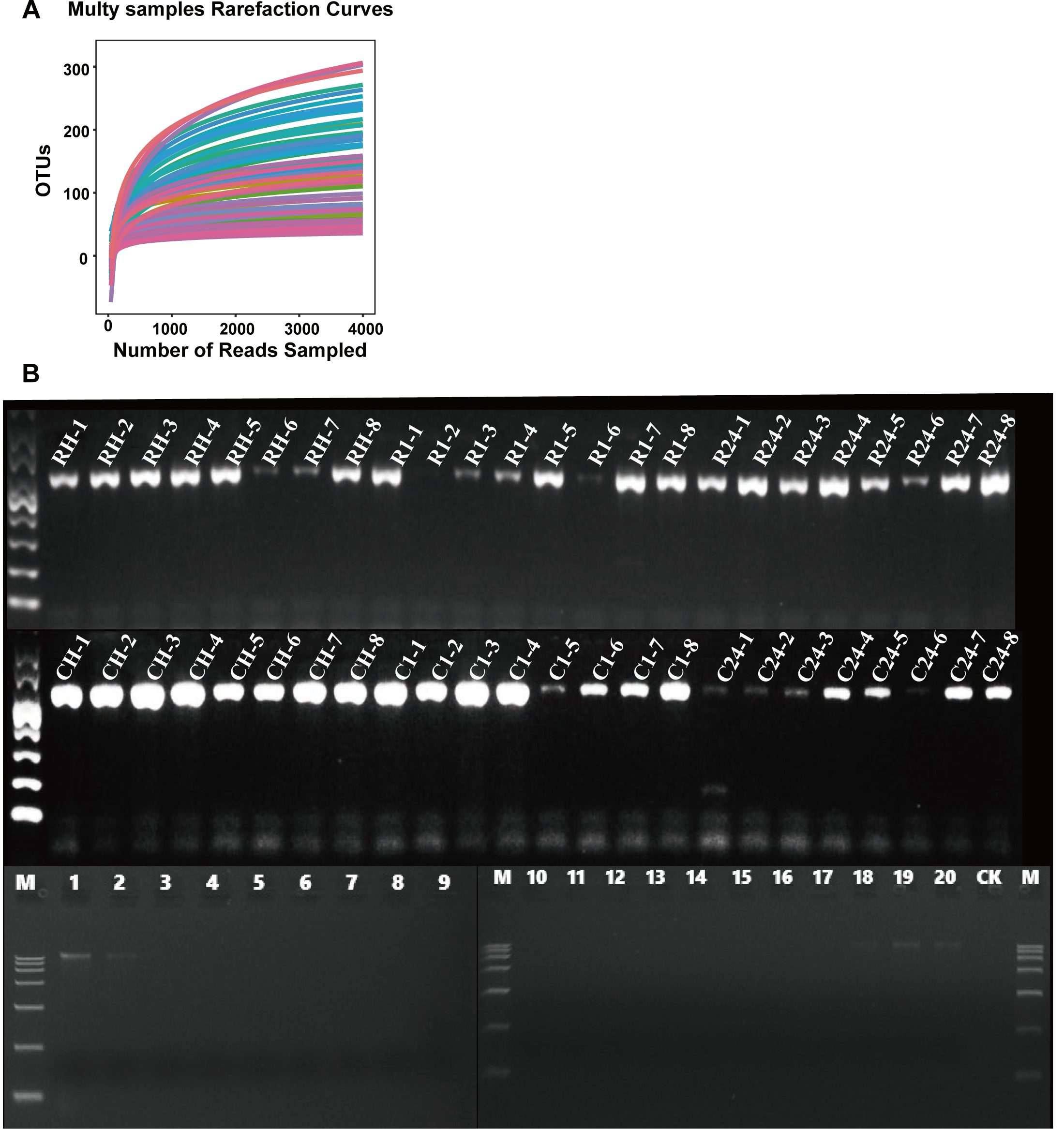

Supplement: Supplementary Figure 1 — Data quality control (A) Rarefaction curves (Sobs index) of the gut microbiota. (B) 16S DNA agarose gel electrophoresis of all samples. RH: retina tissue under normal condition; R1: 1h after laser injury (retina); R24: 24h after laser injury (retina); CH: RPE/choroid tissue under normal condition; C1: 1h after laser injury (RPE/choroid); C24: 24h after laser injury (RPE/choroid); 1-3:sterile cotton swabs sampled from the globe surface before cleaning procedure; 4-5,17:sterile cotton swabs sampled from the globe surface after cleaning procedure; 6-7: lysis buffer; 8-10: 10% betadine; 11-13: sterile saline; 14-16: 70% ethanol. 18-20: positive control. [file Image1.tif]
